# Crystalline Ni_3_C as both carbon source and catalyst for graphene nucleation: a QM/MD study

**DOI:** 10.1038/srep12091

**Published:** 2015-07-14

**Authors:** Menggai Jiao, Kai Li, Wei Guan, Ying Wang, Zhijian Wu, Alister Page, Keiji Morokuma

**Affiliations:** 1 State Key Laboratory of Rare Earth Resource Utilization, Changchun Institute of Applied Chemistry, Chinese Academy of Sciences, Changchun 130022, People’s Republic of China; 2 University of Chinese Academy of Sciences, Beijing 100049, People’s Republic of China; 3 Discipline of Chemistry, School of Environmental and Life Sciences, The University of Newcastle, Callaghan 2308, Australia; 4 Fukui Institute for Fundamental Chemistry, Kyoto University, Kyoto, 606-8103, Japan

## Abstract

Graphene nucleation from crystalline Ni_3_C has been investigated using quantum chemical molecular dynamics (QM/MD) simulations based on the self-consistent-charge density-functional tight-binding (SCC-DFTB) method. It was observed that the lattice of Ni_3_C was quickly relaxed upon thermal annealing at high temperature, resulting in an amorphous Ni_3_C catalyst structure. With the aid of the mobile nickel atoms, inner layer carbon atoms precipitated rapidly out of the surface and then formed polyyne chains and Y-junctions. The frequent sinusoidal-like vibration of the branched carbon configurations led to the formation of nascent graphene precursors. In light of the rapid decomposition of the crystalline Ni_3_C, it is proposed that the crystalline Ni_3_C is unlikely to be a reaction intermediate in the CVD-growth of graphene at high temperatures. However, results present here indicate that Ni_3_C films can be employed as precursors in the synthesis of graphene with exciting possibility.

Graphene, a two-dimensional, single-layer sheet of sp^2^-hybridized carbon atoms, has attracted world-wide attention and research interest, owing to its exceptional physical properties, such as high electronic conductivity, good thermal conductivity, and excellent mechanical strength^1^,^2^,^3^. The past few years have witnessed considerable progress in the scalable, economical growth of graphene, stimulated by its wide potential applications^2^,^3^,^4^. Currently, chemical vapor deposition (CVD) using transition or noble metal catalysts, is the preferred method for high-quality graphene synthesis, due to the scalability and cost-effectiveness of the CVD process^5^,^6^.

Among the substrates used in the CVD process, Cu^7^,^8^ and Ni^9^,^10^,^11^,^12^ have attracted particular attention with contrasting growth properties. In the former case, low carbon solubility of copper leads to self-limiting surface growth and the formation of monolayer graphene without a bulk reservoir effect^7^,^13^. However, the excessive CVD temperature needed for graphene growth, about 1000 °C^13^, results in sublimation of the copper substrate. The as-grown graphene usually displays multiple imperfections such as wrinkles and grain boundaries, due to the weak interaction between graphene and copper^14^. On the other hand, nickel has been found to be a common catalyst for the synthesis of graphitic nanostructures^5^,^15^. Given the fact that the lattice constant of the Ni(111) surface closely matches that of graphene and the strong interaction between nickel and carbon^16^,^17^, nickel was considered to be ideal for epitaxial growth of large homogeneous domains of graphene. Furthermore, the small decomposition barriers of hydrocarbon on Ni and the easy etch of Ni substrate to obtain isolate carbon layer ensure that graphene CVD growth on Ni is one of the promising routes for the synthesis of large-scale graphene^12^,^18^.

Previous studies demonstrated that the following steps are involved in the graphene growth process on Ni-based catalysts: (1) gaseous hydrocarbons are adsorbed and decompose on nickel surfaces; (2) carbon adatoms dissolve into the subsurface or bulk nickel; (3) carbon atoms precipitate back to the nickel surface at low temperature and graphene is formed^12^,^13^. However, the relatively high solubility of carbon in Ni and the resulting bulk reservoir effect^13^ make graphene uniformity and layer control with large areas be a very challenging task on Ni. Recent years have evidenced progress in this aspect. The thickness of catalyst, feedstock concentration, substrate cooling rate and the reaction temperature can be adjusted to make graphene growth on Ni controllable^19^,^20^,^21^,^22^. Nevertheless, growth control remains elusive, despite extensive experimental and theoretical studies aimed at elucidating the graphene growth mechanism^11^,^12^,^23^,^24^,^25^. Significant disparities, however, remain in the scientific communities, particularly with respect to whether the formation of nickel carbide is related to the graphene growth. On one hand, Hofmann *et al.* concluded that bulk crystalline Ni carbide is not present during graphene growth^26^,^27^. On the other hand, metallurgical studies demonstrated the formation of metastable nickel carbide, due to the high carbon solubility of Ni, contributes to the carbon precipitation from Ni^28^. Indeed, metastable nickel carbide has been suggested as an intermediate catalyst phase in graphene growth^9^,^10^,^11^,^29^ and also in carbon nanotube synthesis^30^. Experimental evidence for the transformation between Ni_3_C and graphene was provided by Cao *et al.*^31^. Very recently, a layer of graphene has been observed to precipitate from the Ni_3_C phase accompanied by the decomposition of Ni_3_C, further indicating that Ni_3_C plays an important role during the formation of graphene on nickel catalysts^32^.

In this study, we present quantum chemical molecular dynamics (QM/MD) simulations of graphene nucleation from crystalline Ni_3_C phase. In doing so, we will reveal the atomistic mechanism of this non-equilibrium process and elucidate the relationship between the nucleation of graphene and the crystalline Ni_3_C phase at high temperature.

## Results

### The mechanism of graphene nucleation

**Figure 1:**
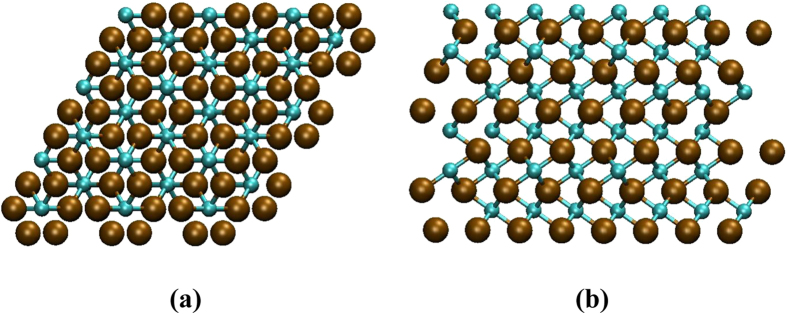
The initial geometry of the crystalline Ni_3_C. (**a**) the top view, (**b**) the side view. Brown and cyan spheres represent Ni and C atoms, respectively.

**Figure 2:**
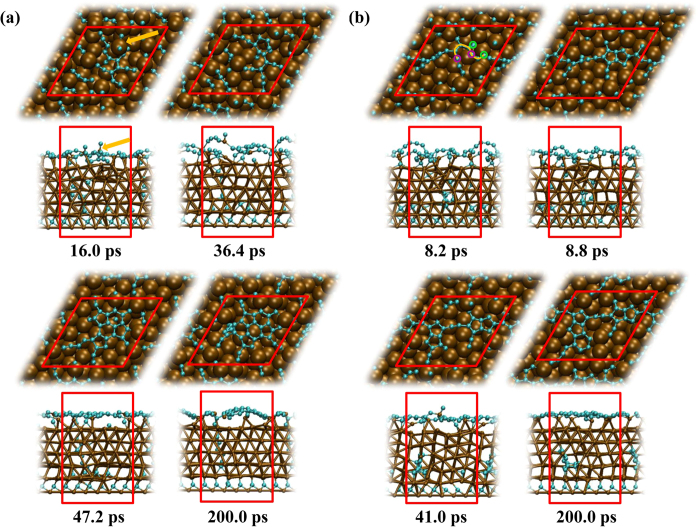
Evolution of graphene nucleation at 1180 K from crystalline Ni_3_C at different simulation time at both top and side views. (**a**) trajectory 3, (**b**) trajectory 5. Color convention is the same as Figure 1. The location of the periodic boundary is indicated by the red line. A surface nickel atom in (**a**) is highlighted by a yellow arrow. The polyyne chain, which exhibited sinusoidal-like vibration in (**b**), is highlighted in yellow line. The two pairs of the bonding carbon atoms in (**b**) are highlighted in purple and green circle, respectively.

The model structure studied in this work is shown in Fig. 1. For details, see Methods section. The snapshots of representative trajectories, i.e., trajectory 3 and trajectory 5, following 200 ps of graphene nucleation simulation, are shown in Fig. 2. They are also detailed in Supplementary Movie S1–S2, respectively. The final structures of trajectories 1–10 are depicted in Supplementary Fig. S1.

It can be seen from Supplementary Movies S1 and S2 that subsurface carbon atoms precipitate out of the catalyst surface at the very beginning of the simulation. Once carbon atoms were separated out, they underwent subsequent diffusion over the nickel surface, which was facilitated by the relatively weak Ni-C interaction compared to C-C interaction. As a natural consequence of the diffusion, the small C_n_ units gradually coalesced with each other to form extended polyyne chains and Y-junctions, which are the prerequisite for the formation of the carbon network. Meanwhile, the Mermin free energy^33^ of the Ni_3_C system drops substantially by *ca.* 3.0 eV per Ni_3_C unit, due to repeated C-C bond formation, making the entire system to be more stable. This minimization of free energy is the driving force for carbon diffusion from bulk Ni_3_C substrate, which is in line with the observations of carbon nanofiber growth^34^.

Figure 2a shows the details of the nucleation process observed in trajectory 3. A complex Y-junction attached with some shorter linear C_n_ chains was formed at 16.0 ps. A hexagon was formed preferentially after ca. 20 ps, which is different from “pentagon-first” mechanism that is usually observed during graphene and carbon nanotube nucleation^23^,^24^. This may be attributed to the role played by the exterior nickel atom that is highlighted by a yellow arrow in Fig. 2a, which indicates the auxiliary effect of the nickel catalyst during the graphene nucleation process. This point will be discussed in further detail below. As growth continued, this structure incorporated more C atoms and shorter C_n_ chains branching out from the original hexagon, and additional graphene rings were added to the nucleus (at 47.2 ps). It is noted that the sp^2^-hybridized structure remained flat throughout this process. Three pentagons and two hexagons were generated sequentially in the immediate vicinity of the original hexagon by the end of the simulation time (at 200 ps). A geometry analogous to the core-shell structured C_21_, which was found to be the precursor in the initial graphene CVD growth^35^, was formed. This precursor became dome-like simultaneously, stabilized by the interaction between its edge carbon atoms and adjacent nickel atoms. This result is consistent with the previous observations^35^,^36^,^37^. An analogous structure was also observed in trajectory 7 (Supplementary Fig. S1).

We will now turn to discuss the reaction observed in trajectory 5 (Fig. 2b), as it shows features more typical, but different from, the mechanism of graphene nucleation in trajectory 3. The rapid precipitation of subsurface carbon atoms and the subsequent formation of polyyne chains preceded the emergence of the “Y-junction” structure, consistent with the aforementioned mechanism. The first sp^2^-carbon polygon driven by the sinusoidal-like vibration of the polyyne chains (highlighted by the yellow line in Fig. 2b), however, became a pentagon under this circumstance at 8.6 ps (Supplementary Movie S2). Carbon nanotube growth shows similar behavior^38^. Following the formation of the first pentagon, the second pentagon was formed immediately through the quick connection between the remaining polyyne chains and the carbon atom extended from adjacent pentagon sites. The two pairs of bonding carbon atoms are highlighted in purple and green circle in Fig. 2b, respectively. This resulted in the formation of a fused pentagon pair (8.8 ps in Fig. 2b), which was anchored to the nickel particles by the carbon branches. Once created, the pentagon-pentagon pair was rather stable and could survive throughout the simulation process. Another fused pentagon pair was formed subsequently (41.0 ps). It is evident that the formation of fused pentagon pairs is a prominent feature of this system (see the snapshots of trajectory 1, 5, 8, 9 and 10 in Supplementary Fig. S1). This result is somewhat unexpected, as it clearly violated the so-called isolated pentagon rule (IPR)^39^, and indicates that this process does not proceed via the most thermodynamically stable pathway. Instead, fused pentagon defects, once formed, become kinetically trapped in the growing graphene structure, and are presumably healed over longer timescales^40^,^41^.

**Figure 3:**
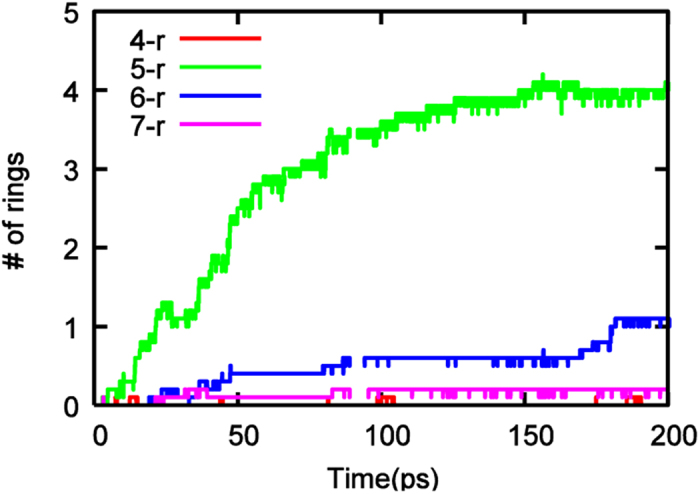
Average polygonal carbon ring populations formed during graphene nucleation from crystalline Ni_3_C.

Figure 3 displays the time evolution of the number of polygon rings during graphene nucleation from crystalline Ni_3_C system, averaged over the ten trajectories. Ring populations of individual trajectory 1–10 are provided in Supplementary Fig. S2. Because the inherent instability of four- and three-membered rings, their formation in the process of graphene nucleation was transitory, and insignificant with respect to the graphene nucleation mechanism. For pentagons, it is found that their numbers increased drastically right after the starting of the simulation process and became the predominant polygon. This is in accordance with the observation of the formation of fused pentagon structures in most of the ten trajectories. The formation of hexagon experienced an induction period and its numbers increased quite slowly with the simulation process. Heptagons were seldom observed, in line with our previous studies in which subsurface carbon atoms segregated out to nucleate a graphene precursor^25^,^42^. It is evidently more favourable for the underlying catalyst structure to be deformed under these conditions, thereby supporting the positively-curved, pentagon-rich structure through Ni-C σ bonding.

**Figure 4:**
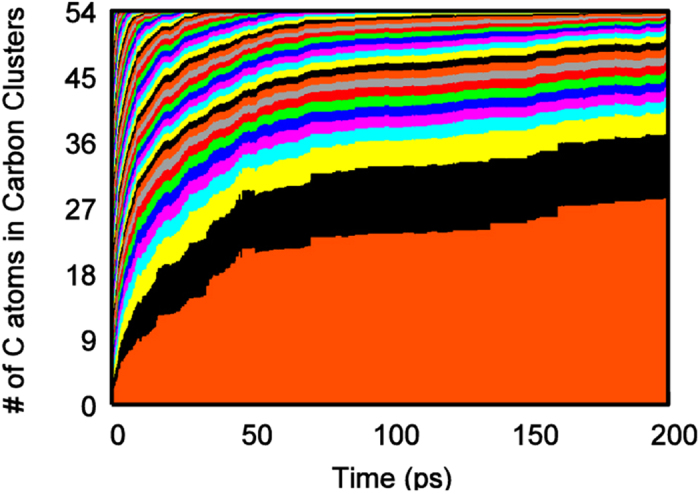
Carbon cluster size (#C_cluster_) evolution in crystalline Ni_3_C as a function of simulation time. Each color indicates a unique carbon cluster observed during the simulation. The way in which the sizes of these colored areas change indicates how these cluster sizes change. In each case, the largest cluster grows by consuming smaller fragments. All data were averaged over 10 trajectories.

The variation in the population of polygons during the simulation process in Fig. 3 is reflected in Fig. 4 accordingly, which shows the time evolution of each individual carbon cluster, averaged in 10 trajectories in terms of size. The applied threshold value for C-C bond lengths is 1.75 Å. The largest cluster grew rapidly in the first 50 ps during which the number of polygons, especially the pentagon, increased promptly (Fig. 3). During this time, the number of the other smaller clusters decreased simultaneously to form large cluster. In the subsequent 150 ps, the increase of polygon slowed down and then reached equilibrium gradually, confirmed by the synchronized change in the size of all the clusters.

### The role of the nickel catalyst

Given the larger strength of C-Ni bond versus that of Ni-Ni bond^43^, nickel layers in Ni_3_C lattice were restricted by the carbon atoms to stay between them to some extent. With the segregation of subsurface carbon atoms, the distance between nickel layers became larger initially, as shown in Supplementary Movie S1–S2. The Ni_3_C lattice was relaxed and damaged rapidly, resulting in a catalyst structure resembled an amorphous Ni_3_C phase. The destruction of the crystalline Ni_3_C structure warranted the high mobility of nickel atoms in the subsequent process, which was visualized clearly in Supplementary Movie S1–S2.

**Figure 5:**
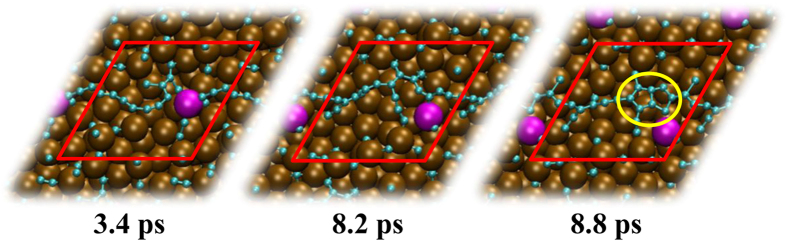
The coalescence of polyyne chains and the subsequent ring condensation observed in trajectory 5 at different simulation time. Color convention is the same as Figure 1. The nickel atom that connects polyyne chains is highlighted in purple. The fused pentagon pair is highlighted in a yellow circle.

With the continuous precipitation of carbon, many nickel atoms were also pulled out. This phenomenon favored the subsequent precipitation of the inner layer carbon atoms by providing larger interspace. On the other hand, the Ni-C_n_-Ni bridge configurations were frequently found to stabilize the polyyne chains on the nickel surface via terminal Ni-C σ bonds. This is consistent with the observation in previous studies^25^,^42^,^44^. Once on the surface, carbon atoms showed high mobility, in spite of obstruction of surface nickel atoms. However, with the connection of carbon atoms on nickel surface, the coalescence of polyyne chains would be subject to a high-energy barrier, due to the obstructive motion of the surface nickel atoms. Interestingly, the coalescence of polyyne chains appeared frequently, ultimately forming carbon networks in the present situation. This is attributed to the mobility of nickel atoms. Figure 5 depicts this process occurring in trajectory 5. Different kinds of C_n_ structures were connected by a single nickel atom on surface at 3.4 ps. Then the nickel atom acted as a “needle”, stitching them together, making them drift on the nickel surface (see the snapshot at 8.2 ps). This is similar to the graphene hole-healing process^45^. Afterwards, the generated branched carbon configurations were less mobile, and coalesced among them by frequent fluctuation. This is understandable, since the branched configurations of carbon have substantial energy barrier for movement even on low-defect nickel surface^46^. As discussed above, fused pentagons were formed promptly (see the snapshot at 8.8 ps). With the formation of carbon networks, the ordering of underlying nickel layers was restored to an extent (Supplementary Fig. S1).

### Comparison between graphene nucleation from crystalline and amorphous Ni_3_C

**Figure 6:**
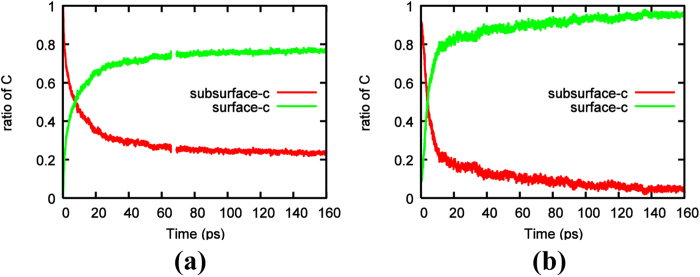
Ratios of subsurface-C/total carbon atoms and surface-C/total carbon atoms as a function of time for (**a**) crystalline Ni_3_C and (**b**) amorphous Ni_3_C^42^, respectively. All data were averaged over 10 trajectories.

The evolution of the number of polygons and the size of carbon clusters during the QM/MD simulations were the product of the movement of carbon atoms in the Ni_3_C system. Figure 6 depicts the variation in the ratios of subsurface and surface carbon to the total carbon, as a function of time in crystalline Ni_3_C and amorphous Ni_3_C systems, respectively. These processes are similar in the changing trend of the ratio of carbon atoms, i.e., a drastic increase in the ratio of surface carbon atoms was accompanied by a simultaneous decrease in the ratio of subsurface carbon atoms. In addition, their equilibrium was reached much earlier (Fig. 6) than that of the number of polygons and the size of carbon clusters (Figs 3 and 4). This indicates that after precipitation on nickel catalysts, different kinds of C_n_ clusters coalesce and contribute to the nucleation of graphene precursor. In light of the rapid decomposition of the crystalline Ni_3_C upon thermal annealing, it is safe to deduce that crystalline nickel carbide is not the reaction intermediate in the CVD-growth of graphene at high temperature. Crystalline nickel carbide observed in experiments^31^,^32^ may be ascribed to graphene production being stopped under the rapid cooling process.

**Figure 7:**
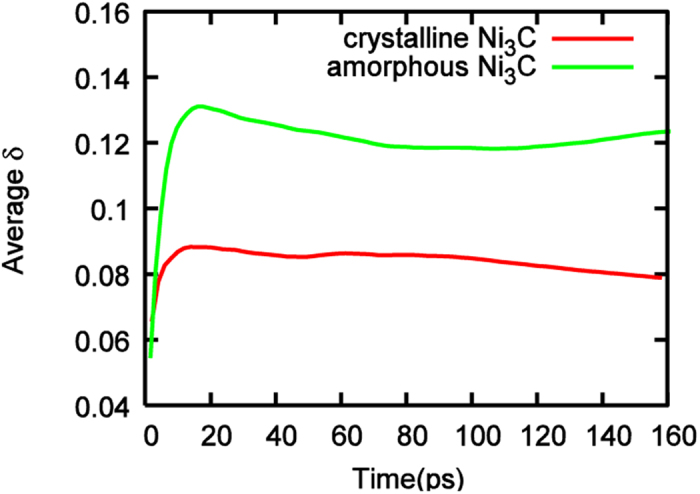
δ values of crystalline and amorphous Ni_3_C at 1180 K. All data averaged over 10 trajectories.

However, the variation of surface carbon atoms increases more slowly in crystalline Ni_3_C compared with the amorphous one. This conclusion is further corroborated by the obtained Lindemann index, δ, for both systems in Fig. 7. It is typically accepted that δ = 0.1 marks the transition between the solid and liquid phases^47^. Figure 7 shows that crystalline and amorphous Ni_3_C catalysts rapidly undergo a solid to liquid-like phase transition upon thermal annealing, as indicated by the rapid increase of δ in both systems. Thus, the atoms in both systems would show high mobility. Nonetheless, the formation of C-C bonds and subsequently the sp^2^-hybridized carbon network on the nickel surface, in part, “solidifies” the system. This can be confirmed by the gradual decrease of the average δ in both systems after the initial peak. The smaller δ for the crystalline Ni_3_C catalyst indicates a more solid-like character that is likely to impede carbon precipitation and graphene nucleation, compared to the amorphous catalyst.

**Figure 8:**
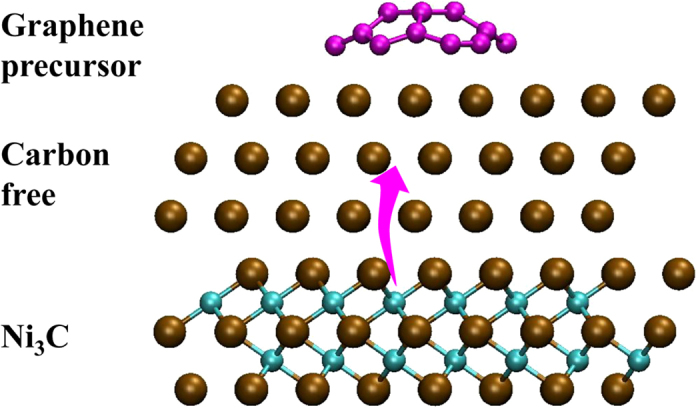
Schematic of graphene growth process from crystalline Ni_3_C . Brown spheres represent Ni atoms. Carbon atoms in deep layer and in nascent graphene precursor are colored in cyan and purple, respectively. Purple arrow indicates the movement (supply) of subsurface carbon atoms.

Within the deeper layers of the crystalline Ni_3_C catalyst, carbon diffusion is slower, presumably due to the greater confining effects of the nickel layers. Under these conditions this subsurface carbon is potentially more likely to gather into subsurface clusters that remain within the catalyst (Supplementary Fig. S1), and do not contribute to the nucleation of nascent graphene precursor. As a result, a carbon-free nickel subsurface layer was embedded in between graphene precursors and bulk nickel carbide (Fig. 8), which has also been found in previous studies^11^,^29^. This phenomenon is different from the previous amorphous Ni_3_C simulation, in which Ni is substituted by C and less layers are selected^42^. This makes the ratio of surface carbon atoms in amorphous Ni_3_C system be larger than that in crystalline one during the simulation process (Fig. 6). This result is also different from the graphene growth starting from elemental Ni catalyst where carbon atoms are added constantly in bulk Ni^25^ and the subsurface carbon clusters precipitate out once reach a certain size. This is because in this study, there is no continual supplement to the inner layer carbon atoms and the subsurface clusters are difficult to segregate out. Therefore, higher subsurface carbon densities are necessary to assist the precipitation of subsurface clusters and the nucleation of ordered graphene precursor on the surface^25^,^42^,^43^.

These results suggest that crystalline Ni_3_C, which includes both carbon source and catalyst, potentially enables facile, controlled graphene synthesis via carbon precipitation. In the initial stage of graphene nucleation, crystalline Ni_3_C can provide carbon source continually. The graphene precursor can be formed by the precipitation of shallow subsurface carbon during the early stages of thermal annealing, as discussed above, which leads to the formation of a carbon-free nickel layer. Subsequently, deeper carbon layers can potentially migrate to this carbon-free nickel layer, which is a thermodynamically controlled process and higher temperature is required (indicated by the purple arrow, Fig. 8). In principle, this process can be repeated continually, opening up the exciting possibility for graphene fabrication by controlling the Ni_3_C thickness and/or the diffusion rate of subsurface carbon atoms.

## Discussion

Graphene nucleation from model crystalline Ni_3_C catalyst surfaces has been studied using QM/MD simulations. The results indicated that the lattice of Ni_3_C was quickly relaxed and damaged upon thermal annealing, resulting in a catalyst structure resembled an amorphous Ni_3_C phase. Inner layer carbon atoms experienced rapid precipitation and subsequent formation of polyyne chains due to the stronger C-C interaction compared with C-Ni interaction. The mobility of nickel atoms played a critical role during the nucleation process. On one hand, it is beneficial to the rapid precipitation of carbon atoms; on the other hand, it enables surface nickel atoms facilitate the coalescence of surface-adsorbed polyyne chains, thereby stabilizing the generated Y-junctions. The frequent sinusoidal-like vibrations of the branched carbon configurations made themselves coalesce to form nascent graphene precursor.

In light of the rapid decomposition of the crystalline Ni_3_C upon thermal annealing, we can deduce that crystalline nickel carbide is not the reaction intermediate in the CVD-growth of graphene at high temperature. Crystalline nickel carbide phases, observed experimentally, may be recovered under the rapid cooling process after the graphene production is stopped. However, this opens up the exciting possibility for experimentalists to fabricate graphene by a proper thickness of Ni_3_C precursor.

## Methods

Graphene nucleation has been investigated in this work with nonequilibrium QM/MD simulations based on the self-consistent charge density functional tight-binding (SCC-DFTB) method^48^. The standard trans 3d-0-1^49^ and mio-0-1^48^ parameter sets were employed. This approach consists of integrating the classical equations of motion, in conjunction with a quantum chemical potential. The nuclear equations of motion of nuclei were integrated using the Velocity Verlet algorithm^50^ (∆t = 1 fs), with the NVT ensemble being maintained via a Nosé–Hoover chain thermostat^51^ (chain-length 3) connected to the degrees of freedom of the system. The nuclear temperature was maintained at 1180 K throughout all simulations, which is the temperature usually employed in experiments^13^,^17^. The quantum chemical potential was evaluated “on-the-fly” at each MD iteration using the SCC–DFTB method, as implemented in the DFTB + program^52^. A finite electronic temperature of 3000 K was enforced throughout all simulations, and so orbital occupations for molecular orbitals near the Fermi energy level were described by a Fermi–Dirac distribution function (and could vary continuously over the interval [0, 2])^33^. The use of a finite electronic-temperature approach alleviated SCC convergence issue, which is from the presence of many near-degenerate Ni d orbitals and unterminated, dangling, C atoms. In such a case, the variational SCC-DFTB energy becomes the Mermin free energy^33^, defined as follows,


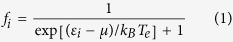



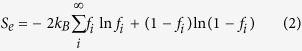



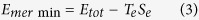


where *T*_*e*_ is the electronic temperature, *f*_*i*_ is the fractional occupation numbers of Kohn-Sham eigenstates (0 ≤ *f*_*i*_ ≤ 1), and *S*_*e*_ is electronic entropy, respectively. This method has been previously applied extensively in the investigations of carbon nanostructure growth^23^,^24^,^25^,^42^.

To our knowledge, two polymorphs of Ni_3_C have been proposed: orthorhombic and hexagonal structures^53^,^54^,^55^,^56^. Previous theoretical studies indicated that the orthorhombic structure is 0.157 eV higher in energy than the hexagonal one per Ni_3_C unit, which was corroborated by experimental observation of the existence of only hexagonal Ni_3_C^53^,^54^. Thus, the hexagonal structure of Ni_3_C with the lattice parameter of a = 4.553 Å and c = 12.92 Å was used in this work^55^,^56^. In the hexagonal structure, the octahedral interstices of nickel atoms are slightly deformed, one-third of which is occupied by carbon atoms. Nickel layers alternate with carbon layers for Ni_3_C (001) surface to form the nuclei of hexagonal Ni_3_C phase. For the QM/MD simulations, a 3 × 3 supercell consisting of Ni_162_C_54_ was employed (Fig. 1). The last layer of Ni atoms and carbon atoms in this model were frozen throughout all simulations, as an approximation to the bulk region. Periodic boundary conditions were enforced using the Γ point approximation, and a vacuum region of 10 nm was applied to avoid interactions between adjacent surfaces. The initial structure, as shown in Fig. 1, was optimized. Then ten independent trajectories were calculated based on the randomized initial velocities, which satisfied a Maxwell–Boltzmann distribution corresponding to 1180 K, for the subsequent graphene growth. These trajectories are denoted using the number 1–10.

The Lindemann index, δ^57^, which was defined in equation (4), was employed here to analyze the physical state of Ni_3_C catalyst.


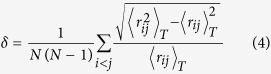


where *N* is the number of atoms in the relevant system, *r*_*ij*_ is the instantaneous distance between atoms *i* and *j*, and the brackets denote thermal averaging over a finite interval of time. The Lindemann index has been used on a number of occasions to elucidate the phase-transitions in transition metal and transition metal carbide nanoparticles, in the context of carbon nanotube nucleation and growth^58^,^59^,^60^.

## Additional Information

**How to cite this article**: Jiao, M. *et al.* Crystalline Ni_3_C as both carbon source and catalyst for graphene nucleation: a QM/MD study. *Sci. Rep.* **5**, 12091; doi: 10.1038/srep12091 (2015).

## Supplementary Material

Supplementary Information

Supplementary Movie S1

Supplementary Movie S2
