# Supplementary material for: Crystalline Ni3C as both carbon source and catalyst for graphene nucleation: a QM/MD study
Source: Sci Rep. 2015 Jul 14;5:12091. doi: 10.1038/srep12091 (PMC4648399; doi:10.1038/srep12091)
Supplement: Supplementary Information [file srep12091-s1.pdf]

Supplementary Information

Crystalline Ni_3C as both carbon source and catalyst for graphene nucleation: a QM/MD study

Menggai Jiao[1,2], Kai Li[1], Wei Guan[1], Ying Wang[1*], Zhijian Wu[1*], Alister Page[3] & Keiji Morokuma[4]

[1]State Key Laboratory of Rare Earth Resource Utilization, Changchun Institute of Applied Chemistry, Chinese Academy of Sciences, Changchun 130022, People's Republic of China

[2]University of Chinese Academy of Sciences, Beijing 100049, People's Republic of China

[3]Discipline of Chemistry, School of Environmental and Life Sciences, The University of Newcastle, Callaghan 2308, Australia

[4]Fukui Institute for Fundamental Chemistry, Kyoto University, Kyoto 606-8103, Japan

*Corresponding authors. Tel: 86-0431-85262801.

E-mail: ywang_2012@ciac.ac.cn (Y.W.); zjwu@ciac.ac.cn (Z.W.)

Movies:

There are two movies showing simulation process and they are provided in separate files.

Movie S1. Evolution of QM/MD simulations of the nucleation of graphene precursor for trajectory 3. Brown and cyan spheres represent Ni and C atoms, respectively.

Movie S2. Evolution of QM/MD simulations of the nucleation of graphene precursor for trajectory 5. Color conventions as in Movie S1.

Figures:

Figure S1. Final structures of trajectories 1–10 following 200 ps QM/MD simulation for the crystalline Ni_3C system. Brown and cyan spheres represent Ni and C atoms, respectively.

Figure S2. Polygonal carbon ring populations of trajectories 1–10 following 200 ps QM/MD simulation.

and C atoms, respectively.

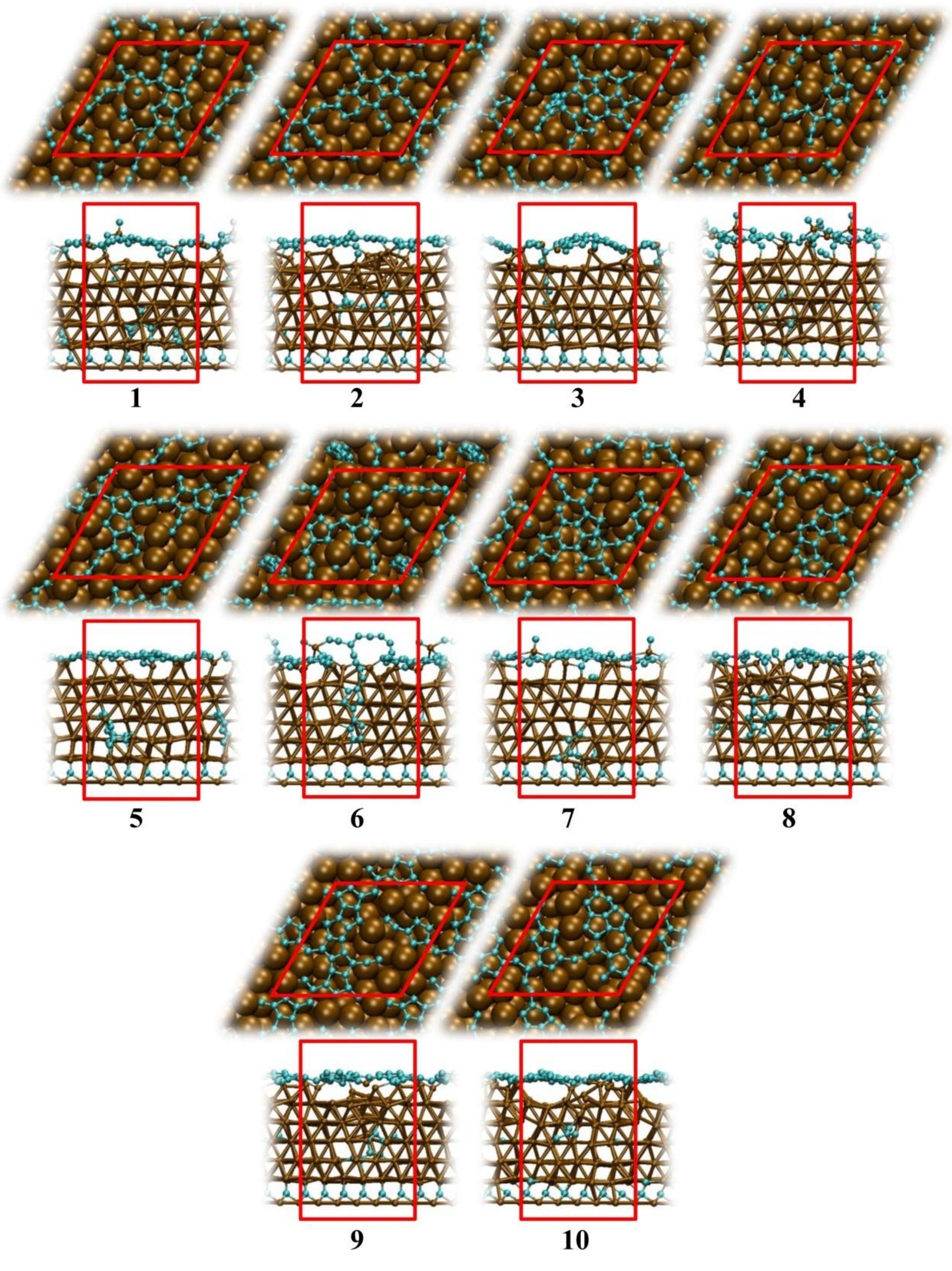

Figure S1 | Final structures of trajectories 1–10 following 200 ps QM/MD simulation for the crystalline Ni_3C system. Brown and cyan spheres represent Ni and C atoms, respectively.

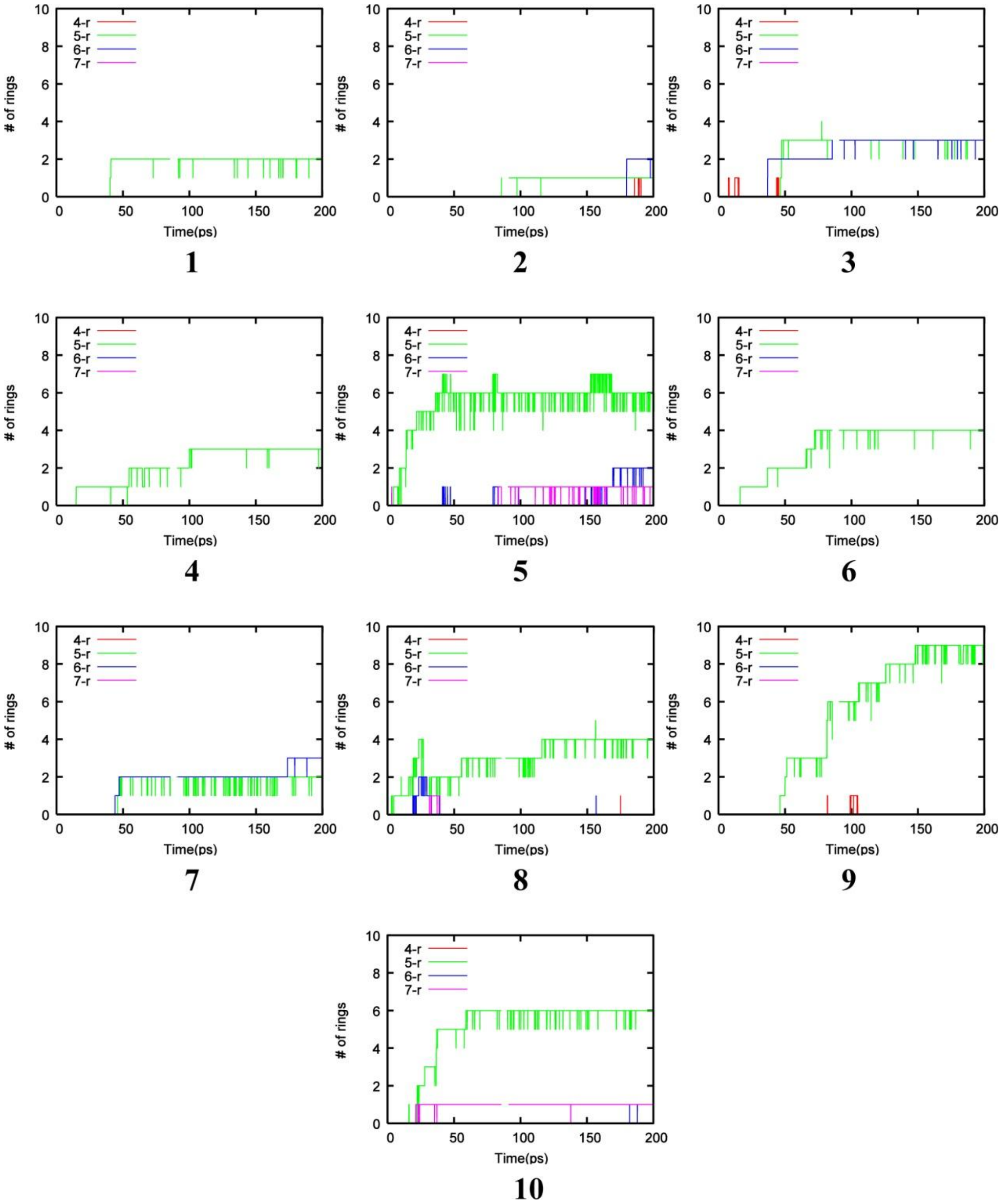

Figure S2 | Polygonal carbon ring populations of trajectories 1–10 following 200 ps QM/MD simulation.
